# Infective Endocarditis in Children as an Increasing Clinical Problem—A Case Series

**DOI:** 10.3390/children11030371

**Published:** 2024-03-20

**Authors:** Urszula Abramczyk, Paweł Cześniewicz, Jacek Kusa

**Affiliations:** 1 Department of Pediatric Cardiology, Regional Specialist Hospital, Research and Development Center, 51-124 Wroclaw, Poland; pczesniewicz@wp.pl; 2 Department of Pediatric Cardiology, Medical University of Silesia, 40-752 Katowice, Poland; jkusa@poczta.onet.pl

**Keywords:** infective endocarditis, guidelines, pediatric

## Abstract

In September 2023, the European Society of Cardiology (ESC) published new guidelines for the management of endocarditis. Infective endocarditis (IE) remains a significant life-threatening disease, concerning an increasingly younger age group, especially children with congenital heart disease (CHD) and young adults after multiple cardiac surgeries. This study’s aim was to alert the medical community to the problem of increasing IE case numbers and IE course complexity. Of the eight patients who suffered from IE treated in 2023 in our department, we describe the four whose course was the most extraordinary. Afterward, we compared the number of IE patients treated over the years in our department. All cases described children with congenital heart disease suffering from IE. The IE clinical presentation in all patients was extremely diverse, necessitating the utilization of all available diagnostic methods. Each child underwent specialized treatment and subsequently qualified for cardiac surgery. While the etiology and treatment of IE are well established, it remains a formidable challenge for physicians. Pediatric patients who have undergone multiple cardiac surgeries constitute a steadily expanding group and are especially susceptible to IE throughout their lives. Currently, no recommendations exist for the management of endocarditis in pediatric patients. This gap compels pediatricians to adapt existing guidelines designed for adult patients and to rely on scientific reports, such as case studies.

## 1. Introduction

In recent years, infective endocarditis (IE) has re-emerged as a progressively significant clinical challenge, impacting increasingly younger patient populations [1,2]. The reasons for this are found not only in increasing bacterial antibiotic resistance but also in a larger number of cardiovascular implanted electronic devices (CIEDs) as well as the greater use of intravenous lines and injection [3,4]. Staphylococcus aureus remains the predominant microbiological pathogen causing IE [5]. As was mentioned above, a change in the age range of individuals with IE has been observed [2]. In the high-risk group, there are patients with prior infective endocarditis, prosthetic valves, any material used for cardiac valve repair, and implanted cardiac devices (permanent pacemakers/implantable cardioverter–defibrillator), as well as patients with congenital heart disease (CHD)—especially cyanotic CHD [1,3]. Diagnosing infective endocarditis remains challenging due to its variable clinical presentation. The clinical IE course is categorized into acute, subacute, and chronic. IE symptoms are heterogeneous—from mild non-fever and non-specific manifestations to serious symptoms such as high fever, valve perforation, myopericarditis, or even stroke. Fever (77.7%), cardiac murmur (64.5%), and congestive heart failure (HF) (27.2%) are the most frequent manifestations of the disease in question [1,6]. IE diagnosis relies on Duke’s diagnostic criteria, modified by the European Society of Cardiology (ESC) [1]. Despite existing effective causative treatment and systematic guidelines being updated, IE morbidity and mortality remain relatively high [1]. At the beginning of September 2023, new guidelines for the management of endocarditis were released by the ESC. This case series features four IE patients’ cases that presented to our clinic before September 2023. The new recommendations meet current needs and fill the knowledge gap since the last guideline update in 2015.

To present IE clinical manifestation complexity, discuss the newest infective endocarditis guidelines released by the ESC in September 2023, and attract the medical community’s attention regarding the problem of the increasing number of IE cases are the main aims of this article.

## 2. Materials and Methods

This study is a retrospective analysis with a descriptive focus, examining a series of four pediatric patients who experienced infective endocarditis (IE) in the Pediatric Cardiology Department in Wroclaw, Poland, in 2023. Patients were selected from the broader group of individuals hospitalized for IE in 2023, specifically those with a challenging course of IE that necessitated collaboration with physicians from various specializations, ultimately culminating in surgical intervention.

Furthermore, this article includes a comparative analysis of the total number of IE cases in the Pediatric Cardiology Department over the past 15 years.

## 3. Case Presentation

### 3.1. Case 1

**Figure 1:**
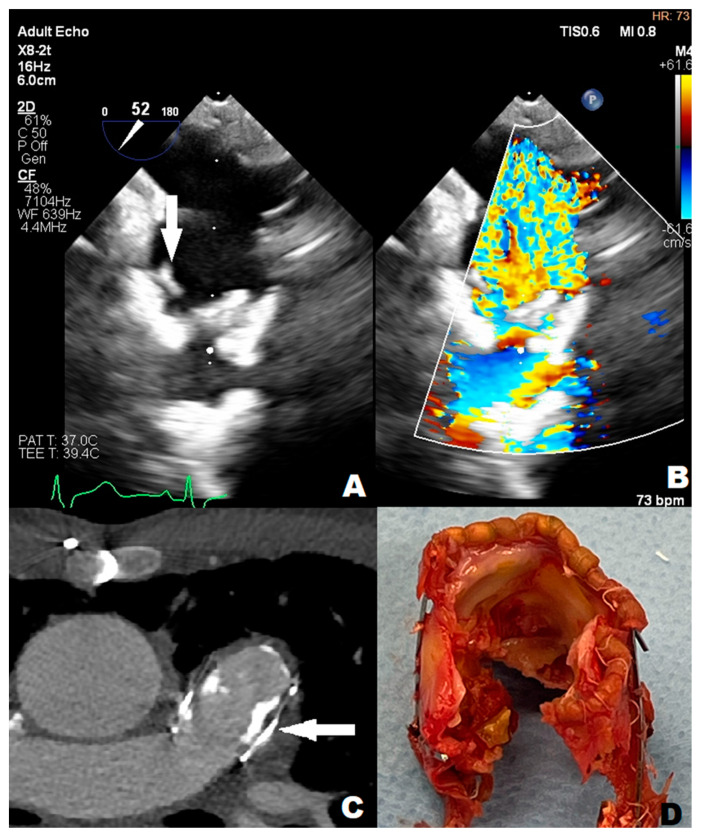
(**A**) arrow point at slightly-built, ballotable vegetation well-visible in TOE (**B**) BioPulmonic regurgitation wave in TOE; (**C**) arrow point at BioPulmonic metal frame visible in angio-CT; (**D**) the BioPulmonic PV after cardiac surgery; TOE—transesophageal echocardiography; angio-CT—angio computed tomography.

An 11-year-old boy was admitted to the emergency department with a 40 degrees Celsius fever and mycotic lesions in the oral mucosa. Previously, the boy had undergone the following procedures: complete repair of Tetralogy of Fallot (ToF) and right pulmonary artery balloon angioplasty during infancy, BioPulmonic 21 biological valve implantation in the pulmonary valve (PV) position at the age of two, and left pulmonary artery reconstruction and balloon angioplasty one year after the reconstruction. The boy did not present other infectious symptoms and his health state was stable. The systolic heart murmur was 4/6 on the Levine Scale (LS). The patient did not present any other symptoms in the physical examination. A series of laboratory blood tests were performed, and the following results were slightly elevated: C-reactive protein (6.30 mg/L) (CRP), procalcitonin (0.10 ng/mL) (PCT), other inflammation indicators, N-terminal pro-b-type natriuretic peptide (319.7 pg/mL) (NT-proBNP), and troponin. The reference ranges of the laboratory tests are provided in the Supplementary Material (Table S1). The inflammatory and congestive changes were present on the chest X-ray. The blood culture samples were taken according to the ESC procedure, which involves obtaining three sets of 10 mL blood samples from peripheral veins at 30 min intervals before initiating antibiotic therapy. Ceftriaxone (2 g/day) and vancomycin were administered empirically for treatment. A few days later, ceftriaxone-sensitive Kingella Kingae (KK) was detected; HACEK group bacteria were isolated from two sets of blood culture samples. The boy was transferred to an upper reference department where ceftriaxone therapy was continued. The TTE examination showed that the right ventricle (RV) was hypertrophied and dilated and the PV flow was turbulent with max PG 120 mmHg with moderate PV regurgitation, without any vegetations. However, the transesophageal echocardiography (TOE) showed numerous, slightly built, ballotable vegetations (Figure 1A,B). The PV cuspids were difficult to see because of the BioPulmonic metal frame and numerous calcifications in the angio-CT (Figure 1C). In summary, the patient met three major criteria and one minor Duke’s criterion, confirming the definite nature of the IE. During the whole period of hospitalization, the boy felt well, the inflammation indicators decreased, and blood culture results became negative, but NT-proBNP (319.7 pg/mL) was still subtly elevated. After the full 6-week treatment, the boy was qualified for PV replacement surgery (Figure 1D). He left the Cardiac Surgery Department in a state of maximum improvement. The patient still remains under outpatient cardiological care.

### 3.2. Case 2

**Figure 2:**
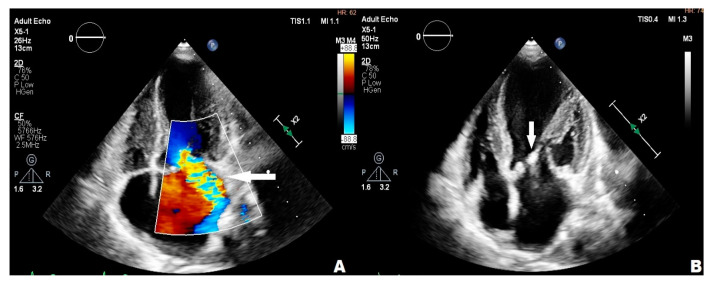
Transthoracic echocardiography; (**A**) arrow point at the mitral regurgitation wave; (**B**) arrow point at hyperechogenic 4 mm × 7 mm lesion in mitral chordae.

**Figure 3:**
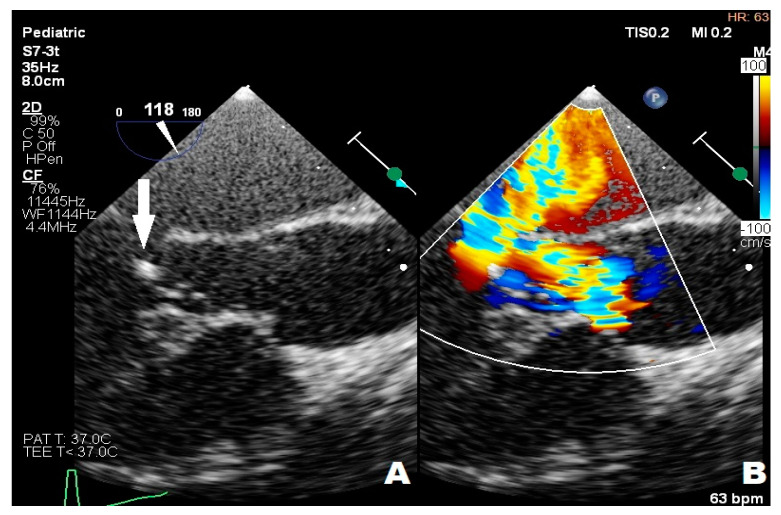
Transesophageal echocardiography; (**A**) the arrow point at vegetation; (**B**) mitral regurgitation wave, aortic regurgitation wave.

An 11-year-old girl with trisomy 21 underwent complete surgical repair for an atrioventricular septal defect (AVSD) during infancy. She presented with thrombocytopenia (65.0 K/μL), petechia, enlarged chest, and abdomen lymph nodes. The diagnostic process was carried out in the hematology department to confirm the Autoimmune Lymphoproliferative Syndrome (ALPS). The vegetations in mitral and aortic valves were found during routine TTE. Any microbiological pathogen was isolated from blood culture samples. The girl did not present any infectious symptoms. A systolic heart murmur of 3/6 in the LS over the cardiac apex and a diastolic heart murmur of 2/4 over the aortic valve were the only abnormalities upon her physical examination. Furosemide (10 mg twice/day), spironolactone (25 mg/day), captopril (12.5 mg/day), teicoplanin (200 mg/day), ceftriaxone (1.5 g/day), and fluconazole (100 mg/day) were administered as the treatment. In the fourth week of her treatment, the patient was transferred to an upper reference department, where further investigation was undertaken. Thickened mitral leaflets, III degree mitral regurgitation, one hyperechogenic 4 mm × 7 mm lesion in mitral chordae, thickened limited-move right coronary aortic valve leaflet (RCL), and severe eccentric aortic regurgitation were found in repeated TTEs (Figure 2A,B), and the transthoracic echocardiography image was confirmed in TOE (Figure 3A,B). In summary, the patient met one major and one minor criterion in Duke’s guidelines for diagnostics, suggesting the possibility of IE. The repeated blood culture samples were negative and the thrombocytopenia (78 K/μL), leukopenia (3.89 K/μL) with neutropenia (0.93 K/μL), mild anemia (RBC 3.8 M/μL), and elevated CRP level (8.4 mg/L) were observed in laboratory tests. The antibiotic therapy was changed in the fourth week of treatment—ceftriaxone was replaced by gentamicin (60 mg/day) due to a persistent elevated CRP level, leukopenia, neutropenia, and anemia, which could be attributed to common side effects of ceftriaxone. During the whole period of hospitalization, the girl felt well, but temporarily elevated creatinine levels, thrombocytopenia, and leukopenia were observed. The girl left the Pediatric Cardiology Department in good condition and with reduced inflammatory indicators in blood tests after the full 6-week treatment, and was qualified for cardiac surgery after full dental treatment and ALPS exclusion.

### 3.3. Case 3

**Figure 4:**
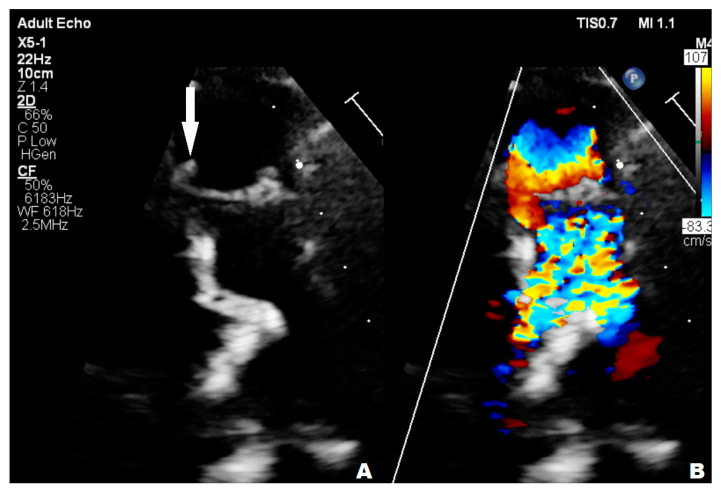
Transthoracic echocardiography; (**A**) the arrow point at ballotable vegetations; (**B**) II degree PV regurgitation; PV—pulmonary valve.

A nearly-16-year-old girl was admitted to the Pediatric Department because of recurrent fever and hepatosplenomegaly. In the past, the girl had undergone procedures such as complete Fallot Tetralogy repair during infancy, Contegra replacement by CorMatrix conduit in pulmonary position during her preschool years, numerous catheter interventions with percutaneous stenting of LPA, and Resilia 23 Edwards implantation two years before her current hospitalization in the Pediatric Department. The patient had suffered from frequent respiratory system infections, recurrent fever, anemia, and microhematuria with cast erythrocytes in urine for the last six months. The teenager did not present other symptoms; however, the clinical examination revealed a systolic heart murmur of 3/6 in LS over the PV, and a significantly enlarged liver and spleen. CRP (20.40 mg/L) and erythrocyte sedimentation rate (45 mm/1 h, 78 mm/2 h) (ESR) were slightly elevated but hemoglobin (7.2 g/dL) (HGB) and erythrocyte mean corpuscular volume (71.0) (MCV) levels were significantly decreased. The treatment started with ceftriaxone (2 g twice/day), then cefixime (400 mg/day) was administered and two units of packed red blood cells were transfused. The patient was transferred to two upper reference departments because of insufficient CRP and the decrease in other inflammation indicators levels and persistent HGB level, where initially the formerly-administered antibiotic therapy was continued. The patient’s general clinical state was stable; the ankles were slightly swollen. Microhematuria with casts erythrocytes, proteinuria (76.9 mg/dL), and elevated levels of protein in 24-h urine (172.50 mg/dL) collection were seen; eGFR was mildly reduced (60 mL/min/1.73 m^2^). Creatinine (1.57 mg/dL), urea (39.0 mg/dL), rheumatic factor (216 Iu/mL) (RF), CRP (20.00 mg/L), lactate dehydrogenase (313 U/L) (LDH), and d-dimer (1893 μg/L) levels were elevated. The antinuclear antibodies (+) (ANA) screening was slightly positive but anti-neutrophil cytoplasmic proteinase 3 antibodies (PR3-ANCA) were highly positive (+++). All the results of the blood culture samples taken according to the ESC procedure were negative. The former antibiotic therapy was replaced with cloxacillin (firstly 2 g, 6 times/day, next 3 g, 4 times/day), ampicillin (firstly 2 g, 6 times/day, next 3 g 4 times/day), and ceftazidime (2 g, 3 times/day). The teenager’s liver and spleen were still enlarged, the kidneys and pelvicalyceal system were in ultrasonographical normal condition, and an insignificant ovarian cyst was visible on abdominal ultrasonography. The TTE examination showed the hypertrophied and dilated RV, reduced RV ejection fraction (EF), enlarged right atrium (RA), reduced IVC respiratory variability, abnormal interventricular septal motion, II degree tricuspid regurgitation with PG 100 mmHg, II degree PV regurgitation with max 36 mmHg, and right ventricle outflow tract (RVOT) stenosis with maximum PG 60 mmHg. The 2.5 mm and 3 mm slightly-built ballotable vegetations were weakly visible on Resilia in TTE (Figure 4A,B), but in TOE all vegetations were well-visible.

**Figure 5:**
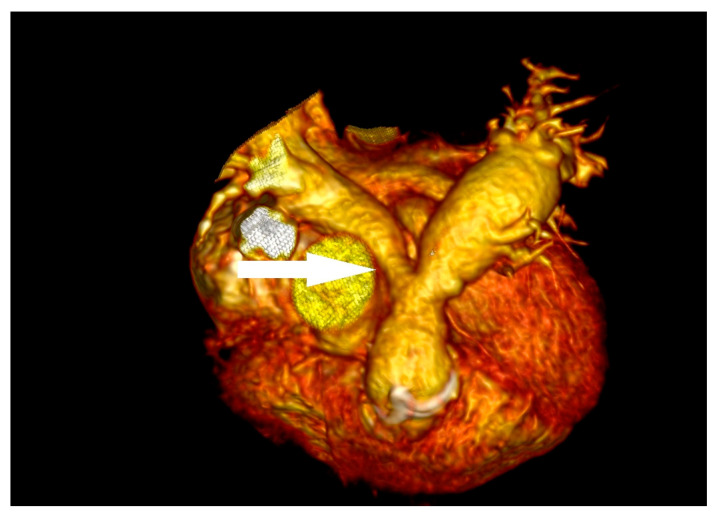
Three-dimensional (3D) model based on MR imaging; the arrow points at mild RPA stenosis; RPA—right pulmonary artery.

**Figure 6:**
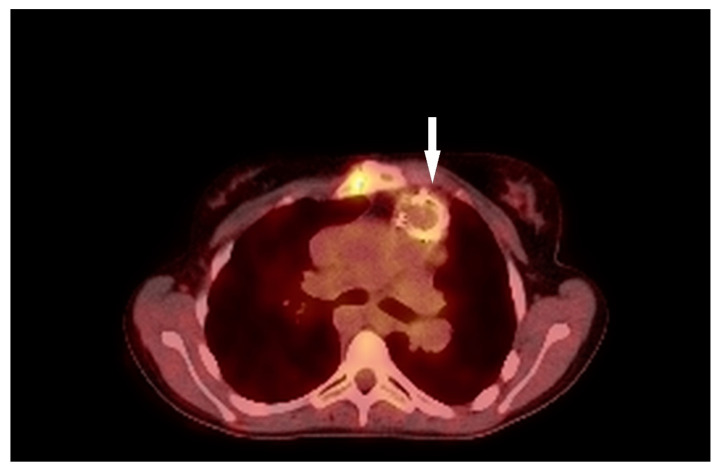
Positron emission tomography/computed tomography; The arrow point at Resilia 23 Edwards metal frame.

Additionally, mild RPA stenosis was seen in a heart MR (Figure 5). The applications of 2-deoxy-2-[(18)F]fluoro-D glucose positron emission tomography/computed tomography (PET/CT) did not show any IE-specific results (Figure 6). Concurrently, granulomatosis with polyangiitis (GPA) was suspected because of microhematuria with cast erythrocytes and positive PR3-ANCA results. The patient met one major and three minor Duke’s criteria, confirming the definite nature of the IE. The paranasal sinus computer tomography showed that the nasal mucosa in Little’s area was thickened. For this reason, methylprednisolone (60 mg/day) was administered for 7 days.

**Figure 7:**
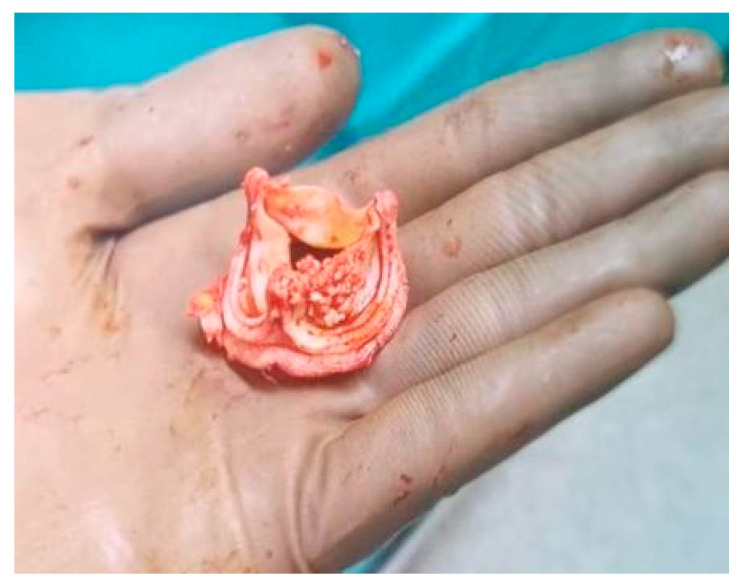
The Resilia 23 Edwards valve after removal.

The ankles’ edemas increased and proteinuria accrued during hospitalization. The red blood cells (3.43 M/μL) (RBC), HGB (7.6 g/dL), and albumin levels (3.57 g/dL) were again significantly reduced; 20% albumin fluid and further units of packed red blood cells were transfused. The 5 methylprednisolone doses (300 mg every 2 days for 9 days) were administered, thanks to which improvements of creatinine level (0.89 mg/dL) and estimated glomerular filtration rate (eGFR) (70 mL/min/1.73 m^2^) were obtained. All other urine and blood test results were persistent. The oligoimmune glomerulonephritis with glomerular crescents was seen upon histopathology examination. The surgery was performed based on the comprehensive clinical assessment of the patient, including an incorrect prosthetic valve, a suspected autoimmune factor, and an inadequate response to the administered treatment. The Resilia 23 Edwards valve was replaced by an aortic valve homograft (Figure 7). The methyloprednisone were replaced by prednisone (40 mg every 2 days). All kidney function markers returned to normal range a few weeks after cardiac surgery. Currently, the patient still remains under outpatient cardiological care in good condition.

### 3.4. Case 4

**Figure 8:**
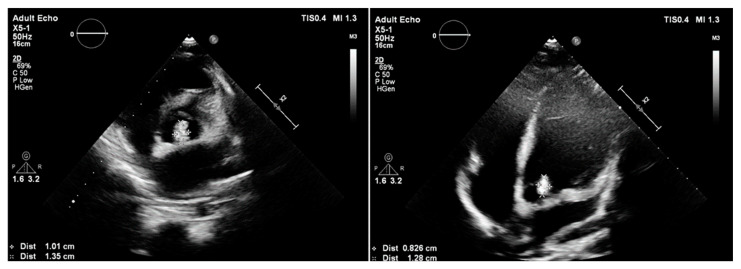
The vegetation in the bicuspid aortic valve before cardiac surgery.

A 14-year-old boy with mild intellectual disabilities was admitted to the hospital because of left spastic hemiparesis, facial nerve palsy, and slurred speech. Apart from neurological symptoms, the clinical examinations revealed a systolic heart murmur of 2/6 in LS. A central nervous system ischemic stroke was confirmed in imaging tests, and the bicuspid aortic valve with ballotable vegetation and moderate regurgitation were found in TTE (Figure 8), subsequently confirmed by TOE.

The CRP level (164.4 mg/L) and other inflammatory indicators were temporarily elevated, which was related to a vascular access inflammatory reaction. All the results of blood culture samples taken according to the ESC procedure were negative. Acetylsalicylic acid (150 mg/day), enoxaparin (40 mg/day), vancomycin (firstly 1.5 g, twice/day for 7 days, next 1 g, twice/day for 10 days), and gentamicin (laboratory antibiotic concentration monitored) were administered as the treatment. In summary, the patient met one major and two minor Duke’s criteria, suggesting the possibility of IE. After nearly four weeks of treatment, papular eruption in the left thigh, hip, and arm and vomiting were observed. Moreover, reduced eGFR (24.90 mL/min/1.73 m^2^) and elevated levels of creatinine (3.59 mg/dL), urea (74.0 mg/dL), NT-proBNP (5762.5 pg/mL), and again CRP (97.40 mg/L) were observed. The antibiotics were replaced by sulfamethoxazole-trimethoprim (480 mg twice/day) and furosemide (20 mg/day for three days) was also administered. Very severe aortic regurgitation and normal left ventricular ejection fraction were obtained from repeated TTEs. Due to the patient’s deteriorating clinical condition, ceftriaxone (2 g/day) was administered in the treatment. The re-performed results of blood culture samples were negative and the patient’s clinical state was stable, making him urgently qualified for cardiac surgery. The native valve was replaced with a mechanical Bicarbon Fitline valve. The histopathology of the native aortic valve showed inflammatory cells infiltration. The patient remains under outpatient neurological, rehabilitation, and cardiological care.

In our pediatric center, we have also noticed that the number of IE cases is growing. In the years of 2008–2018, we diagnosed 15 IE cases. From 2019 to the day of publication, we diagnosed 10 IE cases, where 8 out of these 10 took place in 2022–2023. The characteristics of patients with IE in the pediatric cardiology department between 2008 and 2023 are shown in Table 1.

The data confirm the upward trend described in the latest scientific reports in pediatric patients with CHD. The reasons for this trend are primarily attributed to the increase in the survival rate of patients after corrective cardiac surgery or catheterization procedures, transvenous cardiac implantable electronic devices, or prosthetic heart valve implantations [1,2,3,7]. Other reasons for IE in the pediatric population might also be linked to the increasing number of intravenous injections, antibiotic resistance, patients’ non-compliance, the COVID-19 pandemic, immunosuppression, and the growing popularity of piercings or tattoos, and the removal of skin lesions in inappropriate conditions.

## 4. Discussion

The knowledge gap and numerous new scientific reports published since the last IE ESC guideline forced the ESC group to update the guidelines for IE diagnostics and treatment. The most crucial change seems to concern the definition of IE based on Duke’s diagnostic criteria modified by the European Society of Cardiology (ESC). The new current definition emphasizes the role of imaging examinations in IE—echocardiography (TTE and TOE), cardiac CT, [18F]-FDG-PET/CT(A), and WBC SPECT/CT—which are the major diagnostic criteria.

Unfortunately, there are no specific recommendations or guidelines available for managing endocarditis in pediatric patients. Consequently, pediatricians are compelled to use and align with guidelines created for adult populations with younger patients. The majority of recommendations could be easily adapted to the pediatric group, given the similarities in the development and diagnostic process of IE in both age groups. However, challenges in terms of guideline adaptation may arise due to contraindications, such as the use of certain antibiotics (e.g., doxycycline) in children. In recent years, there has been an observed increase in the number of IE cases, especially in a younger group of patients with cyanotic CHD [2], despite IE being a relatively uncommon pathology in a pediatric population [1,8,9].

All aforementioned patients denied any oro-dental procedures or other invasive diagnostic or therapeutic procedures before the onset the initial IE symptoms. Lifelong antibiotic prophylaxis is recommended and reinforced in the 2023 ESC guidelines for patients with cardiovascular diseases such as previous IE, untreated cyanotic CHD, cyanotic CHD treated with surgery or transcatheter procedures, patients with transcatheter aortic and pulmonary valves, patients with any surgically implanted prosthetic valves or with any prosthetic material used for surgical cardiac valve repair—particularly if oro-dental procedures are anticipated. High-risk patients undergoing an invasive diagnostic or therapeutic procedure involving the respiratory, gastrointestinal, genitourinary tract, skin, or musculoskeletal systems should also consider prophylaxis. Currently, lifelong antibiotic prophylaxis after the oro-dental procedure is recommended for patients with post-operative palliative shunts, conduits, or other prostheses. 

However, antibiotic prophylaxis for patients after surgical repair, with a lack of residual defects or valve prostheses, is only recommended for the first 6 months after the procedure. A new IIa class and level C recommendation for antibiotic prophylaxis in patients undergoing transcatheter mitral and tricuspid valve repair has been introduced. It is crucial for healthcare professionals in all specialties to be well-versed in the updated guidelines and to educate patients and their families because IE prevention measures can safeguard patients from serious health complications.

Total pediatric infective endocarditis concerns only about 10% of children without congenital heart disease [10,11]. IE usually involves structurally incorrect native valves in comparison with structurally well-built native valves. Patients with bicuspid aortic valve (BAV) develop IE more often than the general population [12]. There are numerous case reports describing IE patients with BAV, which is very often accompanied by severe aortic valve regurgitation [13,14,15]. Heart murmurs were described as one of the main clinical symptoms, which also occurred in the aforementioned patients.

Patients with prosthetic valves are in the high-risk group [1] because prosthetic valve endocarditis remains a serious life-threatening problem with a nearly 40% mortality [16,17]. The left-side IE affects bioprosthetic valves much more frequently than mechanical valves [17,18,19]. Two of the previously mentioned patients had left-sided IE and two of them had right-sided IE. Patients with left-sided IE had structurally incorrect native valves; however, patients with prosthetic valves had right-sided IE.

Right-sided IE is rarer than left-sided IE and affects the native tricuspid valve more frequently than the pulmonic valve [20,21]. Isolated pulmonic valve IE is very rare [22], regardless of whether it applies to native or prosthetic valves [20,21]. Some researchers have suggested that the Melody valve is the most prone to developing IE, the Contegra valve is the second in line, and homografts pose the lowest risk [16,23]. None of the two aforementioned patients had one of the earlier listed prosthetic valve types. Despite that, their prosthetic valves in the PV position were their biggest IE risk factor. Patients with intracardiac devices, central venous catheters, and intravenous drug users (IVDU) are particularly vulnerable [1,21]. The rising number of young patients with prosthetic valves and intravenous devices, but also the increased number in the last few years of venous injections and venous catheters during, e.g., fluid therapy, seem to be the most important risk factors in the pediatric group. Patients with CHD after numerous cardiac surgeries are especially IE-prone; patients with prosthetic valves are in the high-risk group, and it is suggested that their diagnostic process should be as fast as possible.

The microbiological etiology might depend on the type of prosthetic valve endocarditis. In a huge retrospective Swedish study, the authors showed that Staphylococcus aureus occurs in mechanical valves the most frequently, whereas α-hemolytic Streptococci (e.g., *S. viridans*) occurs mostly in bioprosthetic valves [19]. The authors of the study emphasized that microbiological etiology could depend on prosthetic valve material [19]. The side of the heart also matters when it comes to microbiological etiology. Right-sided IE is more often caused by Staphylococcus aureus than left-sided IE [20]. Only one of the described patients had a positive blood culture result. The isolated Kingella Kingae (KK) is a gram-negative β-hemolytic coccobacillus belonging to the HACEK group, which is oropharyngeal commensal [24]. Currently, the number of reported cases of IE with KK has slightly increased, and all of the cases have occurred in young-aged children [10,25,26]; the IE caused by KK seems to be induced by improper oral hygiene. The increased number of IE diagnoses may have happened due to the limited access to dentists and health care during the COVID-19 pandemic. That is why the IE may have developed for longer and may have been diagnosed later than in cases before the pandemic.

Blood culture-negative endocarditis poses a clinical challenge and is linked to poorer outcomes compared to cases of culture-positive endocarditis [7,27]. The blood culture-negative IE (BCNIE) can be divided into three groups: (a) BCNIE after antimicrobial treatment, (b) BCNIE caused by microorganisms, which have historically included the HACEK, group, nutritionally variant *Streptococci*, *Pasteurella* spp., mycobacteria, and fungal organisms, and (c) BCNIE caused by intracellular organisms that cannot be cultured in blood using traditional techniques [27]. Currently, the BCNIE may constitute nearly one-quarter of IE cases, but there is a significant disparity among scientific reports [27]. When clinicians consider BCNIE, they should contemplate Liebman–Sacks endocarditis, marantic endocarditis, Behçet’s disease, or even endocarditis in porcine bioprosthetics linked to an allergy to pork [27,28].

In the described cases, blood cultures yielded negative results in three out of the four patients. The blood culture samples were always taken according to the ESC procedure—three sets of 10 mL blood samples from peripheral veins at 30-min intervals —which is important before initiating antibiotic therapy. The obtained laboratory results neither confirmed nor excluded BCNIE during the patients’ observation in the department. The latest 2023 ESC guidelines do not emphasize the use of molecular techniques as the primary IE diagnostic method. Nevertheless, in similar cases, there is room for improvement in the use of more advanced laboratory testing in the future. This approach could result in a faster identification of the causative organism, contributing to accurate management and ultimately leading to improved clinical outcomes. In such cases, empiric antibiotic regimens for BCNIE should be broad, covering a wide spectrum of potential bacterial factors.

IE may manifest itself in various clinical situations. All described cases show a whole range of IE symptoms, from mild to severe. However, despite their quite good general condition, all the described patients finally required cardiac surgery. It must be emphasized that a high clinical suspicion in patients with neurological or glomerulonephritis symptoms—even if the blood cultures are negative—should be assumed to be IE symptoms. Glomerulonephritis in IE usually manifests itself clinically as acute kidney injury and hematuria [29], whereas in histology, mostly as crescentic changes and with diffuse proliferative glomerular changes [29,30]. Moreover, ANCA antibodies have been found in significantly younger patients with IE [30]. An acute ischemic stroke is the most common neurological IE manifestation [31]. Stroke symptoms are easily recognized and not very common in a pediatric population, so physicians should pay special attention to them. All the above-described patients had complicated and unapparent disease courses. They were transferred to upper reference centers in the early stage of IE, which certainly improved their treatment outcomes. Fast diagnosis, efficient treatment, and comprehensive medical care were possible only because of well-cooperating upper reference centers, especially in case 3. Thanks to this, the patients’ quality of life would be improved.

Echocardiography is one of the basic and essential skills in cardiology. Using echocardiography in the first line shortens the diagnostic process. The aforementioned patients’ chests had mostly undergone numerous previous cardiac surgeries, which significantly influenced the transthoracic echocardiography acoustic window. The vegetations were not clearly seen in every case in TTE, which was why TOE always had to be performed. Easy access and its easy performance give TOE a significant superiority over other imaging methods. TOE should be performed in every patient with suspicion of IE after cardiac procedures to shorten the diagnostic path. An early TOE examination might allow for faster treatment administration even if blood culture samples are negative. In all presented cases, treatment was either finished with cardiac surgery or cardiac surgery was planned. The recommendation concerning urgent surgery indications has appeared in the updated IE guidelines. Urgent surgery depends on both vegetation size (≥10 mm) and other surgery indications for all valve types; therefore, the recommendation has I class and C level [1]. The recommendation suggests that urgent surgery may be considered in aortic and mitral valves with vegetation ≥10 mm and without severe valve dysfunction or without clinical evidence of embolism and low surgical risk. Only two cases out of the four described have a hyperechogenic 4 mm × 7 mm lesion in mitral chordae and severe eccentric aortic regurgitation, so the patient was not qualified for urgent cardiac surgery. However, it needs to be emphasized that blood vessel diameter and heart size are obviously dependent on the child’s age. For this reason, the recommendation could not simply be used for all age groups. The z-scores of the vegetation could be one solution for how to use this recommendation. Additionally, PET-CT and CT are not the best choices for imaging prosthetic valves because the prosthetic valve’s metal frame usually produces a lot of artifacts, which can aggravate valve imaging. However, they are fantastic methods for searching for the infection source.

## 5. Conclusions

IE remains a significant life-threatening disease, and its prevalence seems to increase every year despite its well-known etiology, symptoms, highly-developed diagnostic methods, and causative treatment. However, achieving an early diagnosis may be challenging, as blood culture samples are often negative, and polymorphic clinical presentation is not obvious. Pediatric patients with newly recognized glomerulonephritis or stroke should be highly clinically IE-suspected, and pediatric patients who have undergone numerous cardiac surgeries form a continuously expanding group and are particularly exposed to IE throughout their lives. The choice of imaging methods should be tailored to the patient’s profile. There are no recommendations for the management of endocarditis in pediatric patients, forcing pediatricians to adapt the currently available guidelines for adult patients and to rely on scientific reports such as case reports. It is essential to underscore the importance of interdisciplinary collaboration among physicians for efficient endocarditis prevention, early diagnosis, and effective treatment in the pediatric group.

## Figures and Tables

**Table 1:** Characteristics of patients in the pediatric cardiology department in 2008–2013: IE, infective endocarditis; F, female; M, male.

Year	2008–2009	2010–2011	2012–2013	2014–2015	2016–2017	2018–2019	2020–2021	2022–2023
The number of IE patients	4	4	5	2	0	1	1	8
F/M	2/2	1/3	2/3	2/0	0/0	0/1	0/1	3/5

## Data Availability

The data presented in this study are available in the article and in the Supplementary Material.

## Supplementary Materials

The following supporting information can be downloaded at: https://www.mdpi.com/article/10.3390/children11030371/s1, Table S1: The reference ranges of laboratory tests.

## References

[1] Delgado V., Marsan N.A., Waha S., Bonaros N., Brida M., Burri H., Caselli S., Doenst T., Ederhy S., Erba P.A. (2023). 2023 ESC Guidelines for the management of endocarditis: Developed by the task force on the management of endocarditis of the European Society of Cardiology (ESC) Endorsed by the European Association for Cardio-Thoracic Surgery (EACTS) and the European Ass. Eur. Heart J..

[2] Hubers S.A., DeSimone D.C., Gersh B.J., Anavekar N.S. (2020). Infective Endocarditis: A Contemporary Review. Mayo Clin. Proc..

[3] Rajani R., Klein J.L. (2020). CME: Cardiovascular Medicine Infective endocarditis: A contemporary update. Clin. Med..

[4] Baddour L.M., Weimer M.B., Wurcel A.G., McElhinney D.B., Marks L.R., Fanucchi L.C., Esquer Garrigos Z., Pettersson G.B., DeSimone D.C. (2022). Management of Infective Endocarditis in People Who Inject Drugs: A Scientific Statement from the American Heart Association. Circulation.

[5] Cahill T.J., Prendergast B.D. (2016). Infective endocarditis. Lancet.

[6] Habib G., Erba P.A., Iung B., Donal E., Cosyns B., Laroche C., Popescu B.A., Prendergast B., Tornos P., Sadeghpour A. (2019). Clinical presentation, aetiology and outcome of infective endocarditis. Results of the ESC-EORP EURO-ENDO (European infective endocarditis) registry: A prospective cohort study. Eur. Heart J..

[7] McPherson Z., Hariprakash S. (2022). Infant with *Kingella kingae* native valve endocarditis in regional Victoria. J. Paediatr. Child Health.

[8] Eleyan L., Khan A.A., Musollari G., Chandiramani A.S., Shaikh S., Salha A., Tarmahomed A., Harky A. (2021). Infective endocarditis in paediatric population. Eur. J. Pediatr..

[9] Tseng W.C., Chiu S.N., Shao P.L., Wang J.K., Chen C.A., Lin M.T., Lu C.W., Wu M.H. (2014). Changing spectrum of infective endocarditis in children: A 30 years experiences from a tertiary care center in Taiwan. Pediatr. Infect. Dis. J..

[10] Baltimore R.S., Gewitz M., Baddour L.M., Beerman L.B., Jackson M.A., Lockhart P.B., Pahl E., Schutze G.E., Shulman S.T., Willoughby R. (2015). Infective endocarditis in childhood: 2015 update: A scientific statement from the American Heart Association. Circulation.

[11] Le T., Graham N.J., Naeem A., Clemence J., Caceres J., Wu X., Patel H.J., Kim K.M., Deeb G.M., Yang B. (2021). Aortic valve endocarditis in patients with bicuspid and tricuspid aortic valves. JTCVS Open.

[12] Lacalzada-Almeida J., Izquierdo-Gomez M.M., Duque-Gonzalez A., Alonso-Socas M.d.M., Munoz-Rodriguez R. (2022). Infectious Endocarditis Caused by *Pseudomona aeruginosa* on Bicuspid Aortic Valve. J. Med. Cases.

[13] Hou C., Wang W.C., Chen H., Zhang Y.Y., Wang W.M. (2021). Infective bicuspid aortic valve endocarditis causing acute severe regurgitation and heart failure: A case report. World J. Clin. Cases.

[14] Boutaybi M., Doudouh O., Assoweh C.D., El Abbassi O., Ismaili N., El Ouafi N. (2023). Mitro-aortic infective endocarditis on bicuspid aortic valve multicomplicated: A case report. Ann. Med. Surg..

[15] Gierlinger G., Sames-Dolzer E., Kreuzer M., Mair R., Zierer A., Mair R. (2021). Surgical therapy of infective endocarditis following interventional or surgical pulmonary valve replacement. Eur. J. Cardio-Thorac. Surg..

[16] Ivanovic B., Trifunovic D., Matic S., Petrovic J., Sacic D., Tadic M. (2019). Prosthetic valve endocarditis—A trouble or a challenge?. J. Cardiol..

[17] Kahrovic A., Angleitner P., Herkner H., Kocher A., Ehrlich M., Laufer G., Andreas M. (2022). Mechanical versus biological valve prostheses for left-sided infective endocarditis. Eur. J. Cardio-Thorac. Surg..

[18] Berisha B., Ragnarsson S., Olaison L., Rasmussen M. (2022). Microbiological etiology in prosthetic valve endocarditis: A nationwide registry study. J. Intern. Med..

[19] Hussain S.T., Shrestha N.K., Witten J., Gordon S.M., Houghtaling P.L., Tingleff J., Navia J.L., Blackstone E.H., Pettersson G.B. (2018). Rarity of invasiveness in right-sided infective endocarditis. J. Thorac. Cardiovasc. Surg..

[20] Shmueli H., Thomas F., Flint N., Setia G., Janjic A., Siegel R.J. (2020). Right-sided infective endocarditis 2020: Challenges and updates in diagnosis and treatment. J. Am. Heart Assoc..

[21] Chowdhury M.A., Moukarbel G.V. (2016). Isolated Pulmonary Valve Endocarditis. Cardiology.

[22] Van Dijck I., Budts W., Cools B., Eyskens B., Boshoff D.E., Heying R., Frerich S., Vanagt W.Y., Troost E., Gewillig M. (2014). Infective endocarditis of a transcatheter pulmonary valve in comparison with surgical implants. Heart.

[23] Yagupsky P. (2004). *Kingella kingae*: From medical rarity to an emerging paediatric pathogen. Lancet Infect. Dis..

[24] Lenoir M., Desnous B., Raisky O., Vouhé P. (2018). Particular surgical aspects of endocarditis due to *Kingella kingae* with cerebral complication. Interact. Cardiovasc. Thorac. Surg..

[25] Kagan S., Levy I., Ashkenazi-Hoffnung L., Lowenthal A., Goldstein R.E., Landau D., Scheuerman O. (2020). Q Fever and *Kingella kingae* Endocarditis in a Toddler: A Rare Coinfection Case. Pediatr. Infect. Dis. J..

[26] Hunt E.A.K., Somers M.J.G. (2019). Infection-Related Glomerulonephritis. Pediatr. Clin. N. Am..

[27] Takata T., Mae Y., Sugihara T., Isomoto H. (2022). Infective Endocarditis-Associated Glomerulonephritis: A Comprehensive Review of the Clinical Presentation, Histopathology, and Management. Yonago Acta Med..

[28] D’Anna L. (2020). Endovascular treatment of ischemic large-vessel stroke due to infective endocarditis: Case series and review of the literature. Neurol. Sci..

[29] McHugh J., Saleh O.A. (2023). Updates in Culture-Negative Endocarditis. Pathogens.

[30] Loyens M., Thuny F., Grisoli D., Fournier P.-E., Casalta J.-P., Vitte J., Habib G., Raoult D. (2013). Link between endocarditis on porcine bioprosthetic valves and allergy to pork. Int. J. Cardiol..

[31] Kirk F., Vaselli N.M. (2023). Blood culture-negative infective endocarditis: Are we looking hard enough?. Infection.

