# Supplementary material for: Infective Endocarditis in Children as an Increasing Clinical Problem—A Case Series
Source: Children (Basel). 2024 Mar 20;11(3):371. doi: 10.3390/children11030371 (PMC10969324; doi:10.3390/children11030371)
Supplement: Supplementary file 1 [file children-11-00371-s001.zip › children-2895094-supplementary.pdf]

Table S1. The reference ranges of laboratory tests.

The reference ranges for laboratory tests	
**The laboratory test**	**The reference ranges**
Red blood cells (RBC)	4,10 - 5,10 M/ul
Hemoglobin (HGB)	12,0 – 15,5 g/dl
Hematocrit (HCT)	35,00 - 46,00 %
Erythrocyte mean corpuscular volume (MCV)	80,0 - 96,0 fl
Corpuscular hemoglobin (MCH)	26,0 - 32,0 pg
Mean corpuscular hemoglobin concentration (MCHC)	32,0 - 36,0 g/dl
White blood cells (WBC)	4,5 - 10,0 K/ul
Platelet Count (PLT)	140 – 420 K/ul
Erythrocyte sedimentation rate (ESR)	First hour 2 – 12 mm/1h<br>Second hour 4 – 20 mm/2h
Creatinine	0,57 - 1,11 mg/dl
Urea	15,0 - 40,0 mg/dl
Estimated Glomerular Filtration Rate (eGFR)	>90 - normal GFR<br>60 - 89 - mildly reduced GFR<br>30 - 59 - moderately reduced GFR<br>15 - 29 - severely reduced GFR<br>< 15 - kidney failure
protein in 24-h urine collection	< 0,15 g/day
Rheumatic factor (RF)	< 30,0 IU/ml
Antinuclear antibodies (ANA)	+/++/+++
Anti-neutrophil cytoplasmic proteinase 3 antibodies (PR3-ANCA)	+/++/+++
Lactate dehydrogenase (LDH)	125 – 243 U/l
D-dimer	< 500 ug/l
Albumins in serum	3,8 – 5,4 g/dl
C-reactive protein (CRP)	
Procalcitonin (PCT)	< 0,05 ng/ml
Troponin	< 15,6 ng/l
N-terminal pro b-type natriuretic peptide (NT-proBNP)	< 125,0 pg/ml
